# Glypican-3 regulated epithelial mesenchymal transformation-related genes in osteosarcoma: based on comprehensive tumor microenvironment profiling

**DOI:** 10.3389/fimmu.2025.1566061

**Published:** 2025-05-13

**Authors:** Jiaming Zhang, Wei Wang

**Affiliations:** Department of Bone and Soft Tissue Tumor Surgery, Cancer Hospital of Dalian University of Technology, Liaoning Cancer Hospital and Institute, Liaoning, Shenyang, China

**Keywords:** osteosarcoma, epithelial-to-mesenchymal, prognostic model, immune characteristics, gene expression analysis

## Abstract

**Introduction:**

Osteosarcoma (OS) is the most common primary bone malignancy, predominantly affecting children and adolescents. Current treatment approaches have limited efficacy, with a 5-year survival rate of approximately 60%. Epithelial-mesenchymal transition (EMT) plays a key role in the onset, progression, and metastasis of OS, potentially influencing patient prognosis.

**Methods:**

We screened EMT-related genes from multiple transcriptomic datasets of OS and performed unsupervised consensus clustering of EMT-related gene sets. Key EMT-related genes were identified using weighted gene co-expression network analysis (WGCNA) and intersected with differentially expressed genes (DEGs) between OS and normal tissue samples. The least absolute shrinkage and selection operator (LASSO) algorithm was applied to screen candidate genes for developing a prognostic model. Single-cell RNA-Seq (scRNA-Seq) analysis was conducted on OS samples to identify cell populations expressing model genes. Functional validation was performed using si-GPC3 in the MG-63 cell line.

**Results:**

The EMT-based prognostic model demonstrated strong predictive capacity across several validation cohorts. The model effectively predicted immune-related features and immunotherapy responses in high-risk and low-risk patient groups. Seven primary cell types were identified from scRNA-Seq data of OS samples, with the osteoblast population showing the highest proportion of cells positive for model genes. The OS_C3 subpopulation exhibited significantly higher scores and included nine gene modules associated with metabolism, structural integrity, proliferation, differentiation, adhesion, migration, immune responses, inflammatory reactions, and signal transduction. The model genes also demonstrated prognostic value across various cancer types. Knockdown of GPC3 in MG-63 cells resulted in decreased proliferation and migration ability.

**Conclusion:**

This study provides new insights into the potential mechanisms of EMT in OS and its impact on the tumor immune microenvironment and response to immunotherapy. These findings may pave the way for novel personalized treatment strategies for OS patients.

## 1 Introduction

Osteosarcoma (OS) is the most prevalent primary bone malignancy (1), and is more common in children and young people (2). About 5.2 children per million are diagnosed with OS each year (3), and patients 10 to 20 years of age account for about 60% of OS cases (4). The high incidence of OS in adolescents is largely attributed to puberty (1). The 5-year survival rate of OS patients following surgery and chemotherapy is only 60% (5, 6). Several etiological factors of OS have been identified, including chemical agents such as beryllium and methylcholanthrene, physical agents such as radiation, and viruses such as Kaposi’s sarcoma-associated herpesvirus (KSHV) (7). OS originates from mesenchymal cells and can induce osteoblast differentiation, resulting in the formation of malignant bone-like tissues (2). High-grade OS consists of osteoblastic cells, chondroblastic cells, fibroblastic cells, teleangiectatic cells, giant cells, small cells and sclerotic tissue (8), and has been classified into more than 20 histological subtypes. OS is common in the epiphyses of long bones of the limbs, such as the distal femur, proximal tibia, and proximal humerus (5), and frequently metastasizes to the lungs, distant bones and lymph nodes (9). The 5-year survival rate of patients with metastatic OS is only 25%, which reflects worsening prognosis (10). The typical symptoms of OS include pain, local swelling, and limited joint movement. In addition, pathological fractures may occur in a small fraction of patients (11). Currently, primary OS is treated through surgical intervention combined with perioperative neoadjuvant chemotherapy (including high-dose methotrexate, doxorubicin and cisplatin) (12). However, these conventional therapies are often ineffective due to distant metastasis and drug resistance (13). Furthermore, immunotherapeutic strategies such as immune checkpoint inhibitors, adoptive cell transplantation, and cancer vaccines, have limited efficacy (14). Therefore, it is crucial to identify novel therapeutic targets to reach a higher survival rate for patients suffering from OS remains a top priority.

Epithelial-mesenchymal transition (EMT) is process wherein epithelial cells attain the motility and invasive capacity of mesenchymal cells due to changes in the expression of cell adhesion and cytoskeletal proteins (15). EMT plays a key role in embryogenesis, fibrosis, wound healing, inflammation, and cancer initiation and progression (16, 17). In addition, EMT facilitates distant metastasis of tumor cells (18), and may also induce multidrug resistance (MDR) through the ABC transporter ATP binding box (19). However, given the mesenchymal origin of sarcomas, the role of EMT in these tumors may differ from that seen in epithelial tumors. For instance, the overexpression of mesenchymal factors like cadherins can impede OS cells from migrating and metastasizing (20). On the other hand, bone marrow-derived mesenchymal stem cells (BM-MSCs) facilitate the mesenchymal-to-amoeboid transition (MAT) of OS cells by secreting cytokines like IL-6 and IL-8, p thereby enhancing their capacity for migration and invasion (21). Therefore, the role of EMT and mesenchymal-epithelial transition (MET) in OS need further study, especially considering the heterogeneity among sarcoma subtypes. To this end, we screened EMT-related genes from multiple transcriptomic datasets of OS, and constructed a prognostic model using the hub genes. The EMT-based model could predict the prognosis, immune landscape, and immunotherapy response of OS patients across several independent cohorts. Our findings provide new insights into the role of EMT in the progression of OS, along with its possible impact on the tumor microenvironment (TME), which may have implications for the development of more effective treatment strategies.

## 2 Methods

### 2.1 Data acquisition and preparation

Bulk RNA sequencing (Bulk RNA-seq) data was retrieved from the Therapeutically Applicable Research to Generate Effective Treatments (TARGET, https://ocg.cancer.gov/programs/target), Genotype-Tissue Expression (GTEx, www.gtexportal.org/home/index.html), Gene Expression Omnibus (GEO, https://www.ncbi.nlm.nih.gov/geo/), and Tumor Immunotherapy Gene Expression Resource (TIGER, http://tiger.canceromics.org/) databases. The GTEx dataset included 395 normal muscle and bone tissue samples (controls), while the TARGET (n = 88), GSE21257 (n = 53) and GSE16091 (n = 34) datasets included OS samples. In addition, datasets of other tumor types, including Rose2021UC (n = 87), Mariathasan2018UC (n = 298), Liu2019SKCM (n = 121), Braun2020RCC (n = 172), Gide2019SKCM (n = 73) and Van2015SKCM (n = 42), were obtained from the TIGER database. The GSE162454 dataset comprising of single-cell RNA sequencing (scRNAseq) data from six OS patients was retrieved from the GEO database. All data had undergone preprocessing, and the samples with survival duration of 0 days were excluded. EMT-related genes were downloaded from the GOBP_EPITHELIAL_TO_MESENCHYMAL_TRANSITION and HALLMARK_EPITHELIAL_MESENCHYMAL_TRANSITION datasets in Molecular Signatures Database (MSigDB, https://www.gsea-msigdb.org/gsea/msigdb), and from dbEMT 2.0 (http://dbemt.bioinfo-minzhao.org/index.html). Since all data for this study were obtained from public databases, ethical approval was not necessary. The procedures of data collection and analysis adhered closely to the applicable regulations.

### 2.2 Consensus clustering

The EMT-related genes that were common to all three databases were identified using Venn diagram. Unsupervised clustering was performed on these intersecting genes using the R package “ConsensusClusterPlus”. The TARGET-OS samples were categorized into k clusters (where k ranges from 2 to 9), each with distinct gene expression patterns. The optimal number of clusters was determined based on the proportion of fuzzy clustering, the cumulative distribution function (CDF) curve, and the consensus score matrix. The specific criteria are as follows: (1) The maximum k value appears before the inflection point of the proportion of ambiguous clustering (PAC) line graph; (2) The CDF curve is smooth and the downward slope is minimal; (3) The consensus score matrix shows the characteristics of “high cohesion and low coupling”. The optimal clusters were further subjected to dimensionality reduction using Principal Component Analysis (PCA) and t-Distributed Stochastic Neighbor Embedding (t-SNE). The reliability of the selected k value was evaluated by investigating the degree of independence among various clusters in a two-dimensional spatial distribution. The overall survival in the two clusters, and in subgroups stratified by age and clinical stage (I/II and III/IV) were analyzed using the Kaplan-Meier (KM) method. The tumor immune characteristics and infiltration of 30 immune populations in both clusters were evaluated using the ESTIMATE algorithm from the “IOBR” R package using four indicators: tumor purity, immune score, stromal score, and ESTIMATE score.

### 2.3 Weighted gene co-expression network analysis

WGCNA was conducted on the intersecting genes using the “WGCNA” R package to identify the hub genes associated with EMT. A correlation matrix was constructed after excluding the genes with low expression levels or similar expression levels in all samples, and then converted into an adjacency matrix using the power function. The optimal power value (β value) was determined following two conditions: (1) this network is infinitely close to a scale-free network, that is, r^2 is close to 1; (2) connectivity information is retained as much as possible. The Topological Overlap Matrix (TOM) was then constructed to form a co-expression network, and the intersecting genes were hierarchically clustered to form a clustering tree. The modules were classified using the Dynamic Tree Cut method, and those that displayed similar expression patterns were grouped together. The distinct modules were color coded, and their correlation with the EMT clusters was visualized by plotting heat maps. The hub genes were extracted from modules with the strongest connection with the consensus clusters using the following criteria: gene significance (GS) > 0.4 and module membership (MM) > 0.6. The hub genes were functionally annotated by Gene Ontology (GO) analysis, and the most significantly enriched molecular function (MF), cellular component (CC), and biological process (BP) terms were selected.

### 2.4 Construction and validation of the prognostic model

The differentially expressed genes (DEGs) between the OS tissues and para-tumor tissues within the TARGET-OS dataset were screened, and intersected with the module hub genes using Venn diagrams. The candidate model genes independently correlated to the overall survival were identified multivariate Cox regression analysis. LASSO regression was then performed to screen the genes for constructing a prognostic model. The features were selected through the optimal λ corresponding to the smallest binomial deviance. The prognostic model was applied to the training TARGET-OS cohort, and the external validation cohorts GSE21257 and GSE16091. Each cohort was stratified into the high-risk and low-risk groups according to the median risk score, and the survival trends were compared by the Kaplan-Meier method. The predictive ability of the model for 1-, 3-, and 5-year survival was evaluated by the receiver operating characteristic (ROC) analysis.

### 2.5 Multi-omics analysis based on prognostic models

Twenty-one immunomodulatory molecules, including receptors, inhibitory molecules, stimulatory molecules, and chemokines, were retrieved from the TISIDB database (http://cis.hku.hk/TISIDB/). The differential expression of these molecules across risk groups, and between CD8+ T cells, Th1 cells and macrophages within each risk category were analyzed. Additionally, we assessed and quantified the activity levels of seven crucial stages within the anti-cancer immunity cycles across various risk groups, followed by a comparison of the scores presented in a box plot. The CIBERSORT, EPIC, MCP-counter, quanTIseq, TIMER, and xCell algorithms in the “IOBR” R package were used to measure the infiltration of different immune cell types in the two risk groups. The correlation between the EMT model and pathways related to immunotherapy and targeted therapy were also analyzed.

### 2.6 Predictive value of prognostic models in immunotherapy

The Tumor Immune Dysfunction and Exclusion (TIDE, http://tide.dfci.harvard.edu/) database was used to predict immunotherapy response and immune escape in the high-risk and low-risk groups by calculating TIDE scores, CAF scores, Exclusion scores, Dysfunction scores, Myeloid-Derived Suppressor Cell (MDSC) scores, Tumor-Associated Macrophage M2 (TAM.M2) scores, CD8 scores, Gene Expression Profile (GEP) scores, Tertiary Lymphoid Structure (TLS) scores, and Merck18 scores. The predictive model was applied to six real-world immunotherapy cohorts, and the overall survival of the high-risk and low-risk groups in each dataset was determined by the Kaplan-Meier method. Each cohort was divided into the responsive (R) and non-responsive (NR) groups, and the association between the risk score and immunotherapy response was analyzed in each group.

### 2.7 Single-cell analysis

The scRNA-Seq data from GSE162454 was analyzed using the “Seurat” package. The cells with fewer than 300 genes, and those with mitochondrial genes exceeding 10% of the overall expressed genes were excluded prior to dimensionality reduction and clustering. In addition, possible duplicates and lower-order multiplets that appeared during the encapsulation process were also removed, along with cell pairs that remained unsorted during the sample preparation phase. After integrating multiple samples using the “harmony” R package, the UMAP algorithm was used to downscale the filtered dataset. The cells were classified into specific populations based on the “Seurat” package, and their distribution patterns were visualized by plotting a UMAP diagram. The expression levels of 19 cell type-specific markers were measured in the six sample cohorts to determine the relative abundance of the immune cell types. Furthermore, the expression levels of the model genes were also analyzed in the individual cells using the “AddModuleScore” function. Based on the EMT gene expression score, all cells classified as a specific type were divided them into the high-score and low-score groups. The pathways associated with these groups were identified through gene-set enrichment analysis (GSEA). Each cell type was divided into subgroups following a second round of dimensionality reduction clustering. The predicted cellular potency and absolute developmental potential of these subgroups were determined using the cytotrace2 algorithm. To identify the subpopulations associated with EMT, the expression levels of particular model genes were analyzed in individual cells, and the scores of different subpopulations were visualized through UMAP plots.

### 2.8 Co-expression network analysis of single-cell data

The population with highest EMT scores were subjected to hdWGCNA to identify co-expressed cellular modules and hub genes associated with EMT. To create a resilient scale-free network topology, the variations in scale-freeness and connectivity of the co-expression network were examined across different soft thresholds. The cell modules were detected using the dynamic tree cutting technique and the UMAP dimensionality reduction was used to determine cellular distribution and the connections among distinct modules. After calculating the Module Eigengenes (ME) of each module, the hub genes in each module were identified based on the characteristic gene connectivity (KME). Protein-protein interaction (PPI) networks were constructed for each module, and the functional pathways and biological processes associated with the hub genes were derived from the GO-BP 2023 entries and the WikiPathway_2023_Human database.

### 2.9 Pan-cancer analysis

The expression levels of the model genes were analyzed in 18 cancers, and correlation coefficients of each gene pair were calculated. The differential expression of model genes between the tumor and para-tumor tissues in each cancer type by calculating the logFoldChange (logFC), and their prognostic significance in 33 cancer types was determined through Cox analysis. In addition, the expression levels of the model genes were also analyzed in different immune subtypes. Finally, the relationships among the model genes, immune scores, matrix scores, RNA stemness scores (RNAss), and DNA stemness scores (DNAss) were evaluated.

### 2.10 Cell culture and transfection

Human OS cell lines (Mg63) and the human normal osteoblast cell line Nhost were obtained from The Cell Bank at the Chinese Academy of Sciences, and cultured in Dulbecco’s Modified Eagle’s Medium (DMEM) supplemented with 10% fetal bovine serum (FBS; BI, Israel), 100 U/ml of penicillin (HyClone, USA). and 100 µg/ml of streptomycin (HyClone, USA). The cells were maintained in a humidified incubator at 37°C under 5% CO_2_, and passaged every 24 h. The U2OS and 143B cells were transfected with GPC3-specific and control siRNAs (designed and produced by Sangon, China). The cells were harvested using trypsin (KeyGEN, China), washed once with PBS, and seeded into 6-well plates in 2 ml complete medium at the density of 1×10^5^ cells/well. The siRNA construct was mixed with the transfection agent PolyFast (catalog number HY-K1014, MCE, USA) in the specified ratio as per the manufacturer’s instructions. Following incubation at room temperature for 15 minutes, the mixture was centrifuged at low speed for 1 minute and evenly pipetted into the corresponding wells. The culture medium was changed 6 h after transfection, and the experiments were performed 48 h after transfection.

### 2.11 Total RNA extraction and RT-qPCR

The cultured cells were harvested and lysed with Trizol (Takara, Japan) on ice for 5 minutes. Following sequential addition of 200 μl chloroform (SINOPHARM, China), and equal amounts of isopropanol (SINOPHARM, China) and anhydrous ethanol (SINOPHARM, China), the lysate was thoroughly mixed, centrifuged at low temperature, and kept on ice for 15 minutes. The organic phase was removed, and the solution was left to dry for 20 minutes. The RNA precipitate was reconstituted in 20 μl DEPC-treated water, and the concentration was measured using a Nanodrop2000 instrument (Thermo, USA). The RNA was reverse transcribed into cDNA using the PrimeScript RT Kit (TaKaRa, Japan) according to the manufacturer’s guidelines, and the cDNA samples were pre-mixed with the SYBR GreenER Supermix (TaKaRa, Japan) kit. RTqPCR was performed on the 7500 Real-Time PCR System (Thermo Fisher Scientific, USA) following the manual instructions, and the reaction conditions were as outlined in the SYBR GreenER Supermix Kit. The relative expression of GPC3 was calculated using the 2^–ΔΔCt^ method, and normalized to that of β-actin. The primer sequences are as follows: β-actin: Forward: 5’ – CCTGGCACCCAGCACAAT - 3’, Reverse: 5’ – GGGCCGGACTCGTCATAC - 3’; GPC3: Forward: 5’-CG GAATTCCTTGGTGGTGGCGATGCT-3’, Reverse: 5’-TGAAAGGTCGGGATCCCCCGAGGTTGTGAAAGGT -3’. Scrambled siRNA (SCR) and two siRNA duplexes designed to target the GPC-3 gene (21 nt long double-stranded RNA oligonucleotides with dTdT overhangs and sequences as follows: sense, GUGCUUUGCCUGGCU ACAU (dTdT), antisense, AUGUAGCCAGGCAAAGCAC (dTdT)were obtained from Bioneer (Daejeon, Korea). Negative control (NC; si-NC group; 5’-TTCTCCGAACGTGTCACGTTT-3’).

### 2.12 Cell assay of GPC3 in osteosarcoma

By comparing the number and size of different cell clones, the impact of GPC3 on cellular proliferation capacity is evaluated. Total protein is extracted from osteosarcoma cells, and after separation through SDS-PAGE electrophoresis, the proteins are transferred to a PVDF membrane. Immunoblotting analysis is performed using specific antibodies against GPC3 and other proteins related to cell proliferation pathways (such as CTNNB1) to detect the expression level of GPC3 and its effect on relevant signaling pathways. The Transwell chamber is utilized to assess the invasive capacity of osteosarcoma cells. Cell suspension is added to the upper chamber of the Transwell, while the lower chamber contains culture medium with chemokines. After a certain incubation period, the cells are fixed and stained, followed by observation and counting of the number of cells that have migrated through the chamber membrane using a microscope, thereby evaluating the influence of GPC3 on the invasive ability of osteosarcoma cells. Through these experimental methods, we are able to comprehensively assess the function of GPC3 in osteosarcoma cells, including its effects on cell proliferation, clone formation, and invasive capability.

### 2.13 Statistical analysis

Pearson or Spearman correlation coefficients were calculated to evaluate the relationship among variables, depending on the distribution of data. Paired t test was used to compare continuous variables with normal distribution, and the Mann-Whitney U test was employed in case normality was not satisfied. The categorical variables were compared by the Chi-square test or Fisher’s exact test. Survival curves were generated using the Kaplan-Meier method, and the log-rank test was used to determine statistical significance. P-value less than 0.05 was considered statistically significant. All statistical analyses were performed using R software version 4.1.3. Unless stated otherwise, the “ggplot2” package was used to create the graphs.

## 3 Results

### 3.1 Consensus clustering

**Figure 1:**
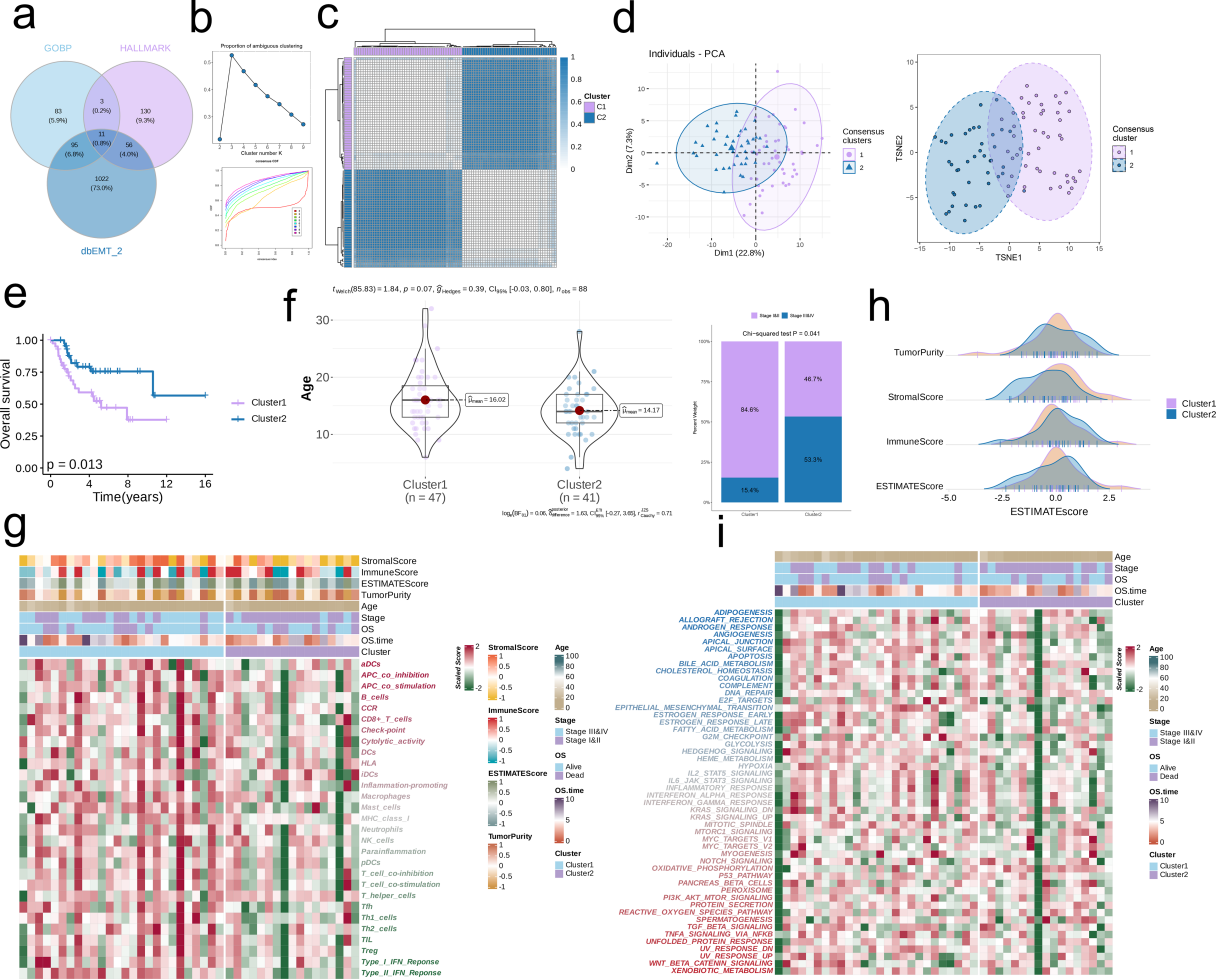
Consensus clustering by EMT-related genes. **(a)** Consensus EMT-related genes acquired from GO_BP, HALLMARK, and dbEMT 2.0. **(b)** The PAC score for each k (indicated by colors). The CDF curves of the consensus matrix for each k. **(c)** The consensus score matrix of all samples when k = 2. A higher consensus score denotes higher similarity. **(d)** PCA and t-SNE analysis. **(e)** Survival analysis of the EMT-related clusters C1 and C2. **(f)** The distribution of age and clinical stages in C1 and C2. **(g)** Immune cell infiltration in C1 and C2. **(h)** Tumor Purity, Stromal Score, Immune Score, and ESTIMATE Score in C1 and C2. **(i)** HALLMARK-related pathways in C1 and C2.

As shown in the Venn diagram in **Figure 1a**, eleven EMT-related genes were common to the GOBP, HALLMARK and dbEMT2.0 databases. Consensus clustering of these genes (k =2) revealed two sample clusters (C1 and C2) in the TARGET-OS dataset (**Figures 1b, c**). As shown in the PCA and TSNE plots in **Figure 1d**, the two clusters were independent. Furthermore, C1 and C2 had distinct prognostic profiles and clinical characteristics. C1 was associated with lower OS (p = 0.013, **Figure 1e**) and older age compared to C2. In addition, the proportion of clinical stage I & II cases (84.6%) exceeded that of stage III & IV cases (15.4%) in C1, whereas C2 had a higher proportion of individuals diagnosed with stage III&IV OS (53.3%) compared to those with stage I&II tumors (46.7%) (p = 0.041, **Figure 1f**). Furthermore, C2 exhibited lower immune infiltration compared to C1, which is indicative of an immunosuppressive TME (**Figure 1g**). The immune score and ESTIMATE score of C2 were higher, and the stromal score was lower compared to that of C1. On the other hand, there was no significant difference in the tumor purity of the two clusters (**Figure 1h**). The downregulation of HALLMARK-related pathways was more obvious in C2 (**Figure 1i**).

### 3.2 WGCNA

**Figure 2:**
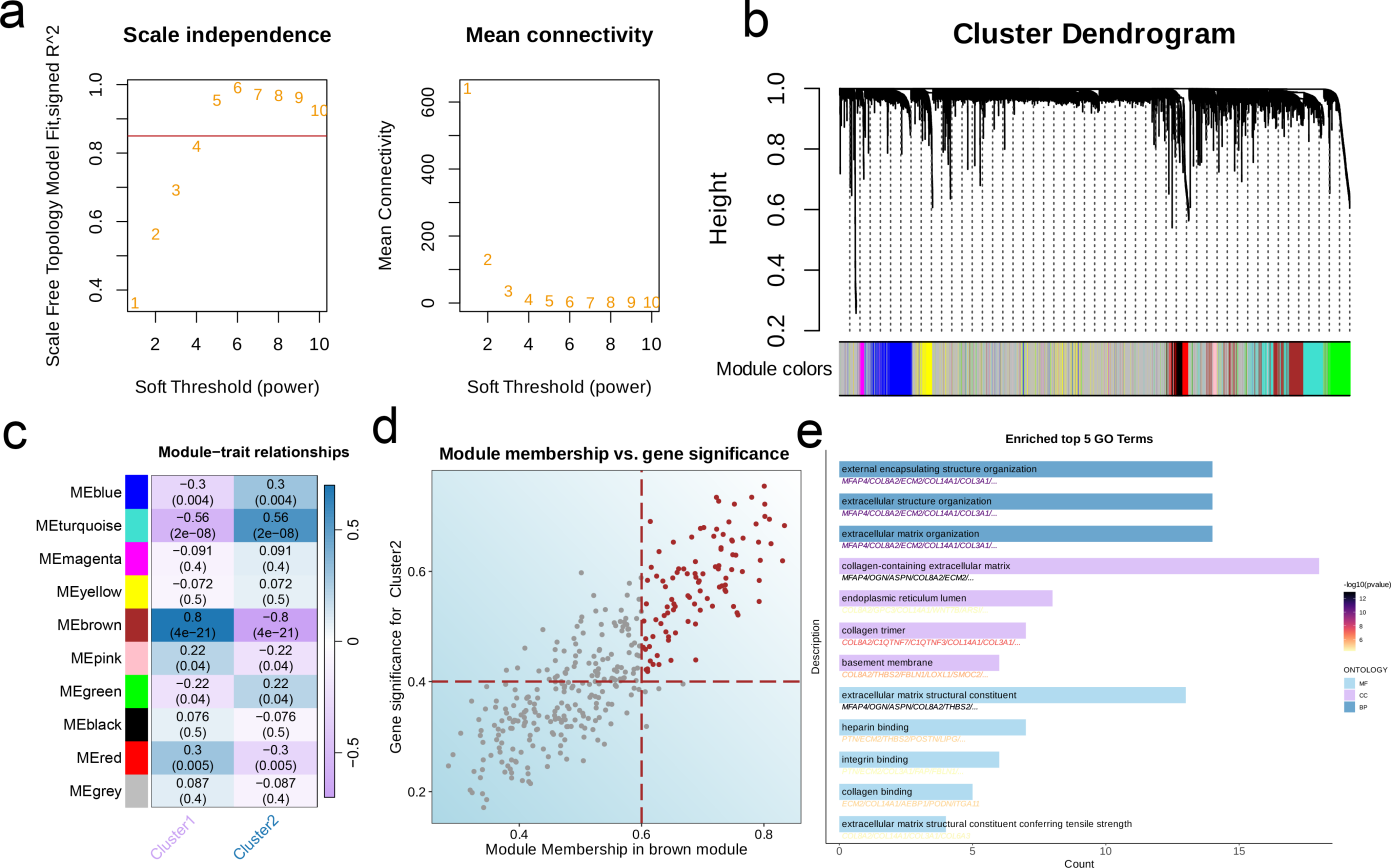
Characteristic genes in EMT clusters. **(a)** Network topology for different soft-threshold power. The left panel shows the impact of soft-threshold power (power = 5) on the scale-free topology fit index; the right panel displays the impact of soft-threshold power on the mean connectivity. **(b)** Cluster dendrogram of the co-expression modules. Each color indicates a module. **(c)** Module-trait heatmap showing the correlation between module eigengenes and EMT clusters. **(d)** Correlation between module membership and gene significance in the brown module. Colored dots indicate the hub genes (MM > 0.6 & GS > 0.4). **(e)** Top five enriched GO terms of hub genes.

By analyzing the scale independence and average connectivity results, we selected β = 4 as the optimal soft threshold, at which the network exhibited good scale-free properties and appropriate connectivity (**Figure 2a**). After dynamic cutting and merging, 10 different gene modules were generated (**Figure 2b**). The brown module showed the strongest correlation with C2, and had a total of 101 hub genes (p < 0.001, r = -0.8, **Figures 2c, d**). The top enriched GO terms for the hub genes were extracellular matrix (ECM) organization, extracellular matrix structural constituent, extracellular matrix structural constituent conferring tensile strength, collagen-containing extracellular matrix, basement membrane, and other extracellular matrix. In addition, pathways related to heparin binding, integrin binding, collagen binding, external encapsulating structure organization, extracellular structure organization, endoplasmic reticulum lumen, and collagen trimer showed significant enrichment (p < 0.001, **Figure 2e**).

### 3.3 Construction and validation of the prognostic model

**Figure 3:**
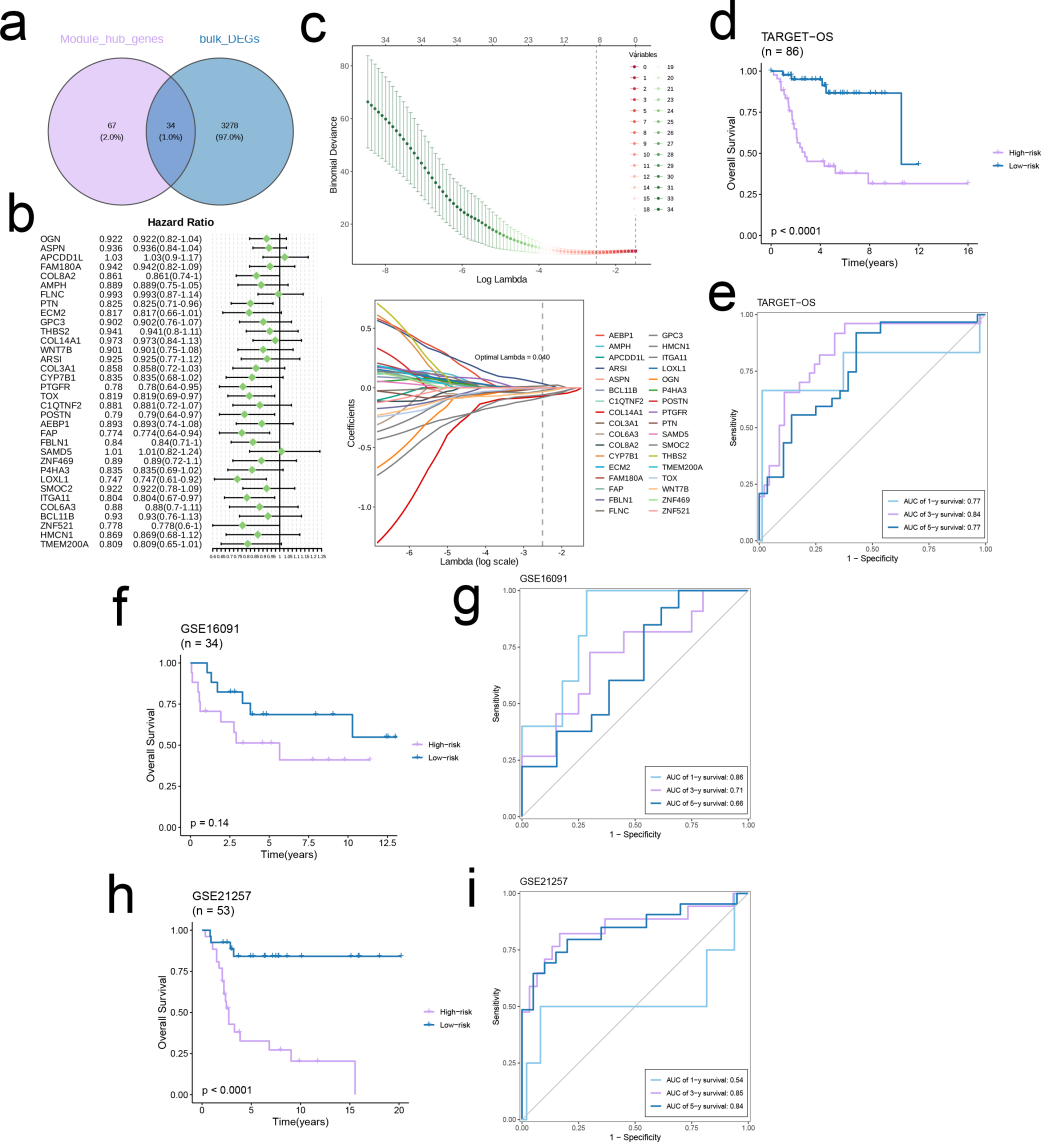
Construct a robust EMT signature. **(a)** Venn diagram of module hub genes and DEGs from TARGET-OS bulk cohort. **(b)** Univariate cox regression analysis of 34 genes in TARGET-OS cohort. **(c)** The selection of prognostic genes based on the optimal parameter λ in the LASSO regression analysis. **(d-i)** Kaplan-Meier curves showing the survival outcomes of patients in the two risk groups in three cohorts. Time-dependent ROC curves were drawn to assess 1-, 3-, and 5-year survival in the three cohorts.

There were 34 intersecting genes between the hub genes and DEGs in TARGET-OS (**Figure 3a**), of which PTN, PTGFR, TOX, POSTN, FAP, LOXL1, and ITGA11 were identified as independent prognostic protective factors for OS (Hazard Ratio < 1) in the multivariate regression model (**Figure 3b**). To minimize the binomial deviance, we selected the λ corresponding to the lowest point of the cross-validation curve, i.e., λ = 0.04, and further extracted the model genes. Seven genes, including COL3A1, FBLN1, FAP, GPC3, CYP7B1, ECM2, and AMPH, were used to construct the prognostic model (**Figure 3c**). The prognosis of the high-risk group was significantly worse than that of the low-risk group across three independent cohorts. Furthermore, the area under the ROC curve (AUC) of the prognostic model for 1-, 3-, and 5-year survival rates were all > 0.6, indicating good predictive ability (**Figures 3d–i**).

### 3.4 Immune-related analysis based on the prognostic model

**Figure 4:**
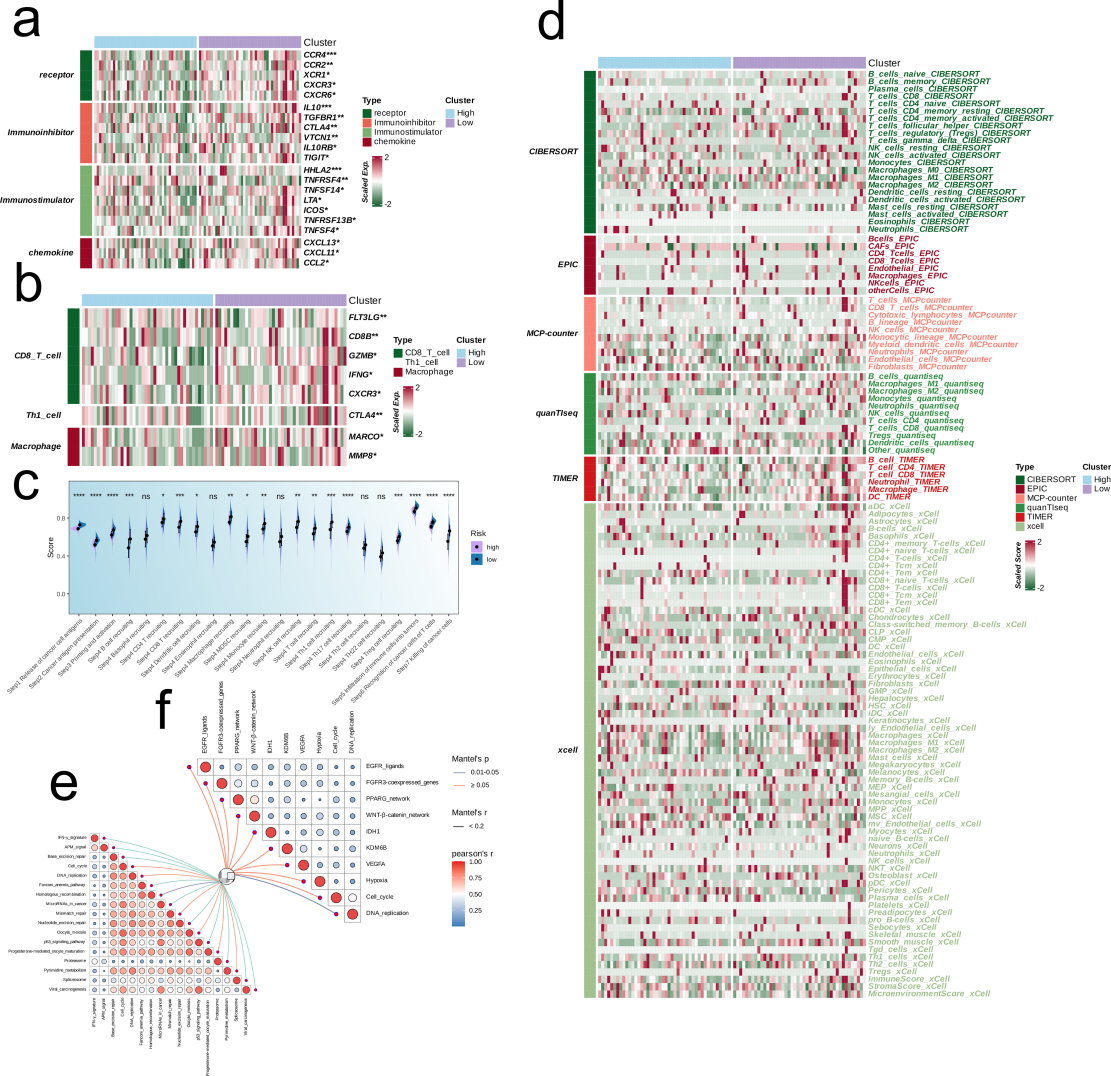
Multi-omics analysis based on EMT signature. **(a)** Heatmap showing the correlation between EMT signature and immune-related molecules. **(b)** Heatmap showing the correlation between EMT signature and immune cell-related effectors. **(c)** Boxplot showing the scores of anti-cancer immunity cycles between low-risk and high-risk groups. **(d)** Heatmap showing the difference between the EMT signature and immune infiltrating cells via TIMER, QUANTISEQ, CIBERSORT, MCPCOUNTER, XCELL, and EPIC algorithms. **(e, f)** Butterfly plots showing the correlation of EMT signature with immunotherapy-associated pathways **(e)** and target therapy-associated pathways **(f)** in TARGET-OS.

Given the variations in the expression levels of immune-related genes between the risk groups, we performed a targeted analysis of immunomodulatory molecules, including receptors, inhibitory molecules, stimulatory molecules, and chemokines. The genes related to anti-tumor effector immune cells (CD8 T cells, Th1 cells and macrophages) were upregulated in the low-risk group, whereas the high-risk group had a higher abundance of immunosuppressive genes (p < 0.05, **Figures 4a–d**). In addition, the prognostic model was correlated to pathways involved in immunotherapy and targeted therapy (**Figures 4e, f**).

**Figure 5:**
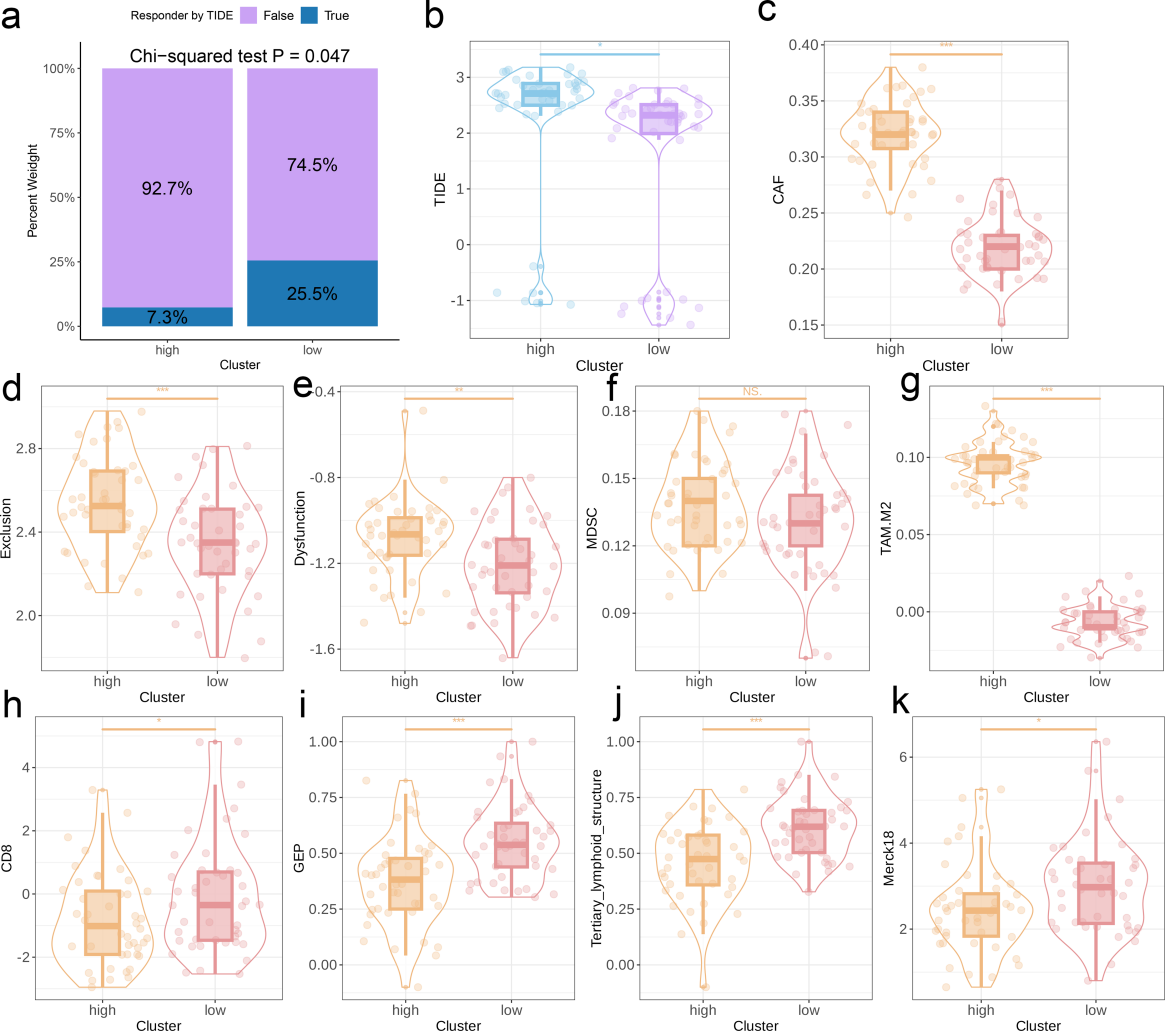
Predictive value of the EMT signature for immunotherapy response. **(a)** Distribution of immunotherapy responders in the EMT clusters as predicted by the TIDE. **(b-k)** Box plots showing the TIDE score, CAF score, Exclusion score, Dysfunction score, MDSC score, TAM-M2 score, GEP levels, CD8 score, TLS levels, and Merck18 score between two EMT score groups.

**Figure 6:**
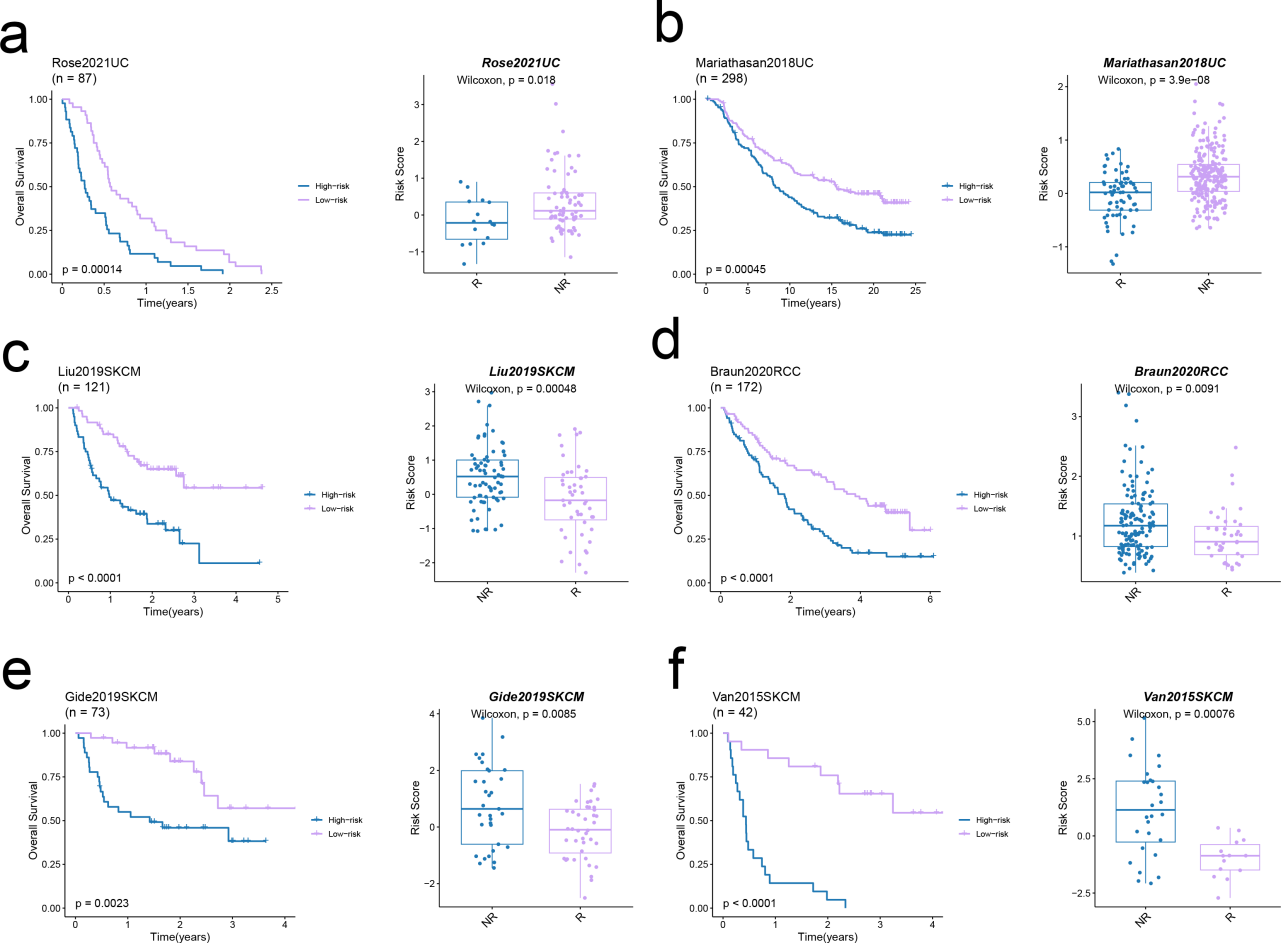
Predictive value of the EMT signature in real-world immunotherapy cohorts. **(a-f)** Kaplan–Meier curves showing the overall survival in low-risk and high-risk groups in six immunotherapy cohorts (left panels). The Box plots illustrate the link between risk score and response in these cohorts (right panels).

The TIDE algorithm was used to compare the immunotherapy response in the risk categories. As shown in **Figure 5a**, 92.7% of the patients in the high-risk group and 92.7% in the low-risk group were classified as non-responders to immune checkpoint blockade. The overall percentage of non-responding patients was 74.5% (see **Figure 5a**). The TIDE score (**Figure 5b**), CAF score (p < 0.001, **Figure 5c**), Exclusion score (p < 0.001, **Figure 5d**), Dysfunction score (**Figure 5e**), and TAM.M2 score (p < 0.01, **Figure 5g**) in the high-risk group exceeded that in the low-risk group, which was indicative of greater immune evasion in the former. Increased CAF infiltration in the high-risk group is consistent with poor prognosis, whereas higher Exclusion score signifies more pronounced rejection of immune cells and suboptimal response to immunotherapy, and high Dysfunction score is indicative of the functional suppression of tumor-specific T cells. Increased infiltration of MDSCs and TAM-M2 in the TME may enhance the immunosuppressive effect and lower the efficacy of immunotherapy. However, the infiltration of MDSCs was similar in the two risk groups (**Figure 5f**). In contrast, the scores for CD8 T cells, GEP, TLS, and Merck18 were higher in the low-risk group (p < 0.05, **Figures 5h–k**), suggesting enhanced immune activity which is likely linked to an improved response to immunotherapy and a more favorable prognosis. We applied the prognostic model on six independent, real-world immunotherapy cohorts, and observed significantly worse prognosis in the high-risk group compared to the low-risk group. In addition, the NR group had a higher risk score than the R group, indicating that the EMT-based model can the prognosis and immunotherapy response in OS patients to a certain extent (p < 0.001, **Figures 6a–f**).

### 3.5 Single-cell analysis

**Figure 7:**
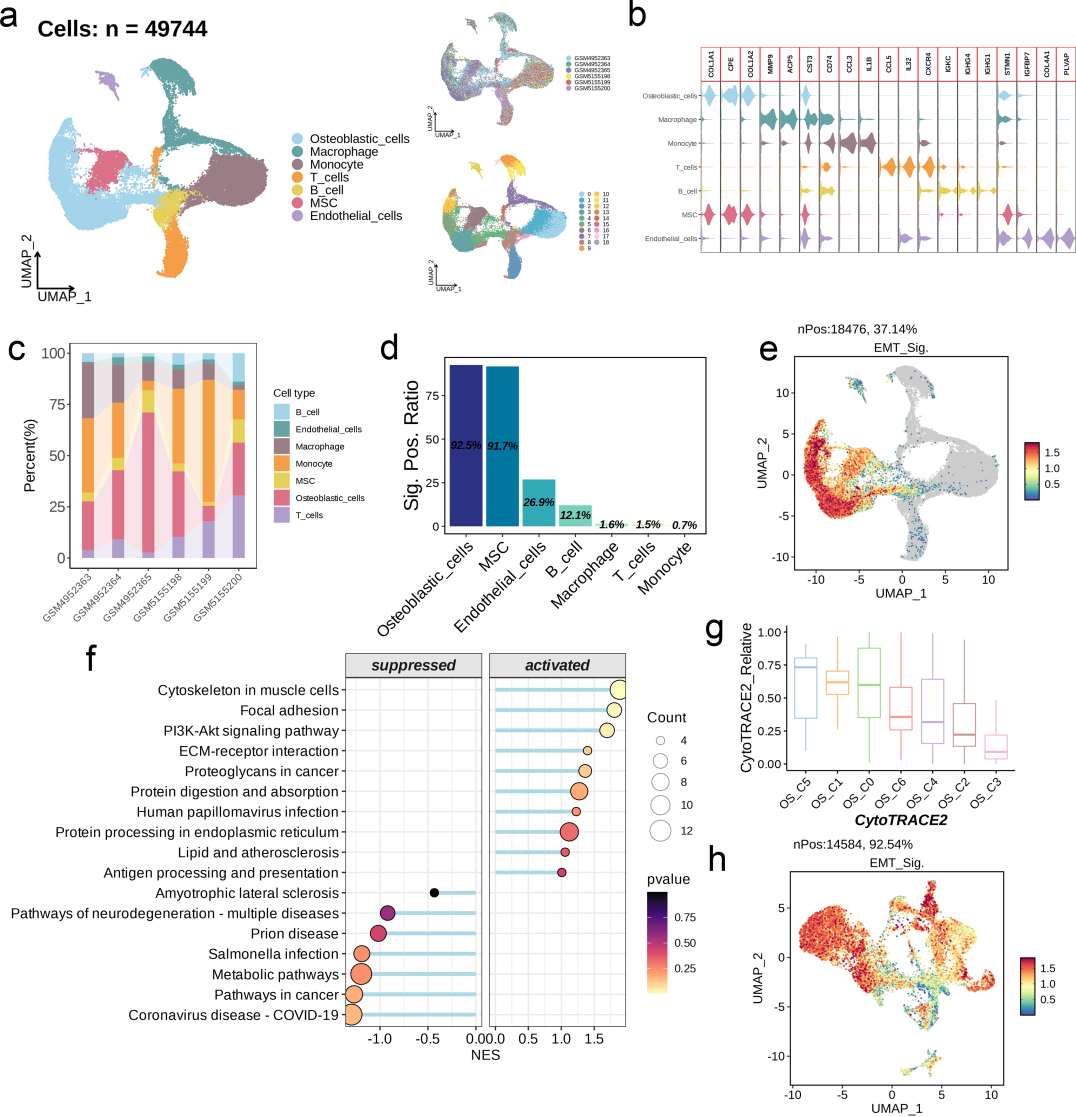
Single-cell analysis of EMT signature. **(a)** UMAP visualization of OS scRNA-seq data. **(b)** Violin plots showing the markers of each cell type. **(c)** Abundance of cell types across different samples. **(d)** The positive ratio of EMT signature in each cell type. **(e)** Distribution of the signature scores across all cell types. The signature score was calculated by the *AddModuleScore()* function implemented in the Seurat package based on the genes derived from the prognostic model. **(f)** GSEA reveals significantly altered pathways in osteoblastic cells with high signature scores compared to those with low scores. **(g)** Boxplots showing the predicted cellular potency and absolute developmental potential of the seven osteoblastic cell subsets. **(h)** Distribution of the signature scores across the seven osteoblastic cell subsets.

After quality control and sample de-batch integration, 49,744 cells were retained and used for single-cell analysis. We identified 19 distinct populations at a resolution of 0.6, which were classified into seven main categories, including osteoblastic cells, macrophages, monocytes, T cells, B cells, mesenchymal stem cells (MSC), and endothelial cells (**Figure 7a**). The osteoblastic cells and MSCs share markers like COL1A1, CPE, and COLA1A2 (**Figure 7b**). As shown in **Figure 7c**, the proportion of these different cell types varies significantly among the six data sets. Furthermore, the model genes were expressed in 92.% of the osteoblastic cells and 91.7% of the MSCs, which significantly exceeded that for other cell types (**Figure 7d**). The distribution of the risk score in different cell types followed similar trends (**Figure 7e**). GSEA of osteoblastic cells showed upregulation of pathways related to cytoskeleton in muscle cells, focal adhesion, and Pl3K-Akt signaling in the high-scoring cells, while metabolic and cancer-related pathways upregulated in the low-scoring population (**Figure 7f**). The UMAP algorithm was applied again to osteoblastic cells, and seven subpopulations were identified (OS_C0 to OS_C6). To further explore the possible developmental trajectories of osteoblastic cells, we analyzed the differences in differentiation potential among these subpopulations. OS_C3 had the lowest differentiation potential, indicating that these cells are likely in the terminal stage of development. On the other hand, OS_C5 had the highest differentiation potential and are likely osteoblastic stem cells (**Figure 7g**). Interestingly, the risk score was markedly increased in the OS_C3 population (**Figure 7h**).

### 3.6 Co-expression network analysis of single-cell data

**Figure 8:**
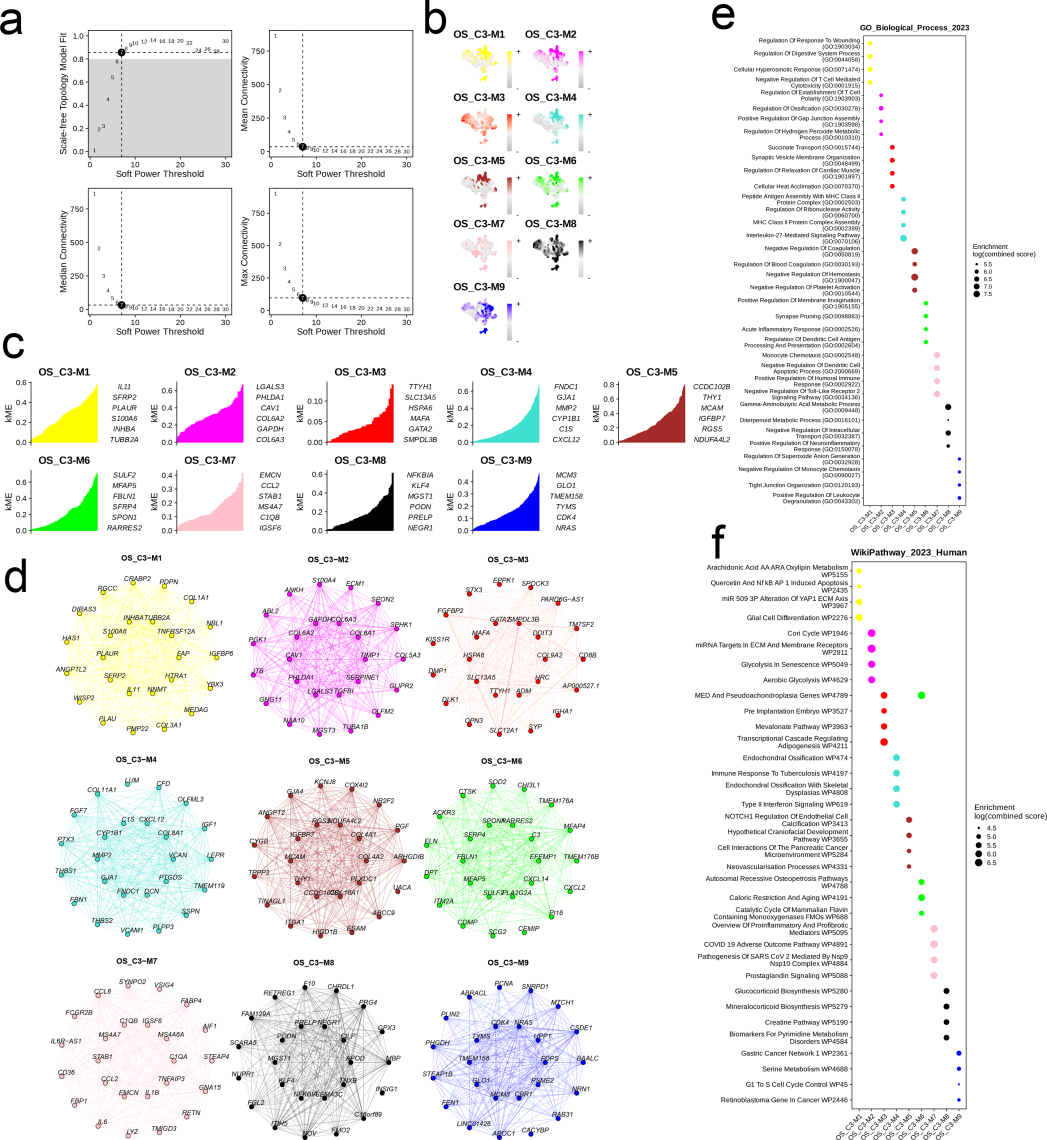
The cell module identified by hdWGCNA method in cells with high EMT signature. **(a)** Analysis of network topology for different soft-threshold power. A power of 7 was select for as the optimal threshold. **(b)** UMAP of the identified 9 cell modules of the OS_C3 cells. **(c)** The hub genes of each cell module. **(d)** The PPI network of the hub genes of each cell module. **(e, f)** The top six enriched GO_BP terms and WikiPathways of the hub genes in each cell module.

We developed a co-expression network based on single-cell data after determining 7 as the ideal soft threshold (**Figure 8a**). As shown in the UMAP diagram in **Figure 8b**, there were nine cell modules with unique distribution characteristics (**Figure 8b**). The hub genes in the nine modules were as follows: OS_C3-M1 - IL11, SFRP2, PLAUR, S100A6, INHBA, TUBB2A; OS_C3-M2 - LGALS3, PHLDA1, CAV1, COL6A2, GAPDH, COL6A3; OS_C3-M3 and OS_C3-M4 - FNDC1, GJA1, MMP2, CYP1B1, C1S, CXCL12; OS_C3-M5 - CCDC102B, THY1, MCAM, IGFBP7, RGS5, NDUFA4L2; OS_C3-M6 - SULF2, MFAP5, FBLN1, SFRP4, SPON1, RARRES2; OS_C3-M7 - EMCN, CCL2, STAB1, MS4A7, C1QB, IGSF6; OS_C3-M8 - NFKBIA, KLF4, MGST1, PODN, PRELP, NEGR1; OS_C3-M9 - MCM3, GLO1, TMEM158, TYMS, CDK4, NRAS (**Figure 8c**). The PPI network of the hub genes in different modules is shown in **Figure 8d**, and their functional pathways are outlined in the bubble plot in **Figures 8e, f**.

### 3.7 Pan-cancer analysis of model genes

**Figure 9:**
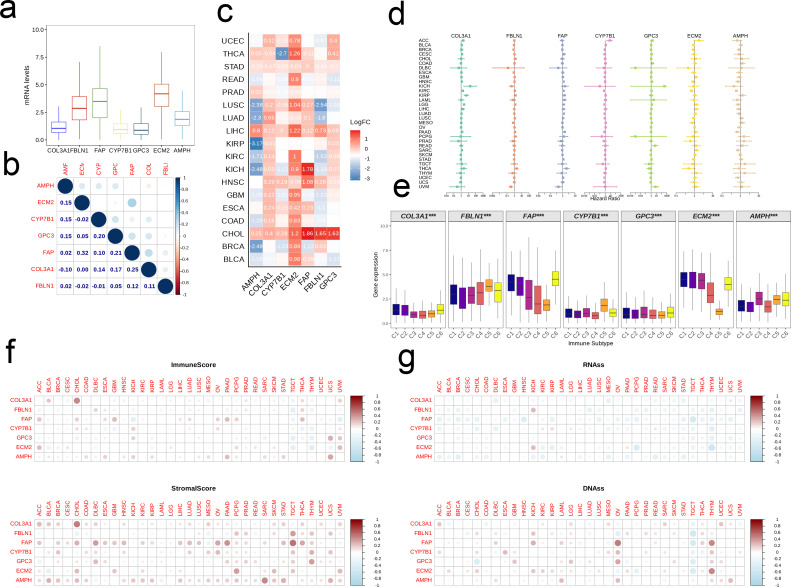
Pan-cancer analysis of the EMT signature. **(a)** Boxplot showing the pan-cancer expression levels of the signature genes. **(b)** The correlation between signature genes. **(c)** Heatmap showing the differential expression of signature genes between tumor and normal tissues in each cancer type. **(d)** Forest map showing the correlation between EMT signature and patient prognosis on a pan-cancer scale. **(e)** Box plot showing expression levels of signature genes in different immune subtypes. **(f, g)** Association of EMT signature with the Estimate Score, Immune Score, DNAss and RNAss in multiple tumors.

The expression levels of seven model genes ranked from high to low were as follows: ECM2, FAP, FBLN1, AMPH, COL3A1, CYP7B1, and GPC3 (**Figure 9a**). While most of the genes were positively correlated, only GPC3 and FAP showed positive correlation with all model genes. In addition, the relationship between FAP and ECM was most significant (r = 0.32, **Figure 9b**). ECM2 was elevated in tumor tissues of the majority of cancer types, whereas AMPH was frequently demonstrated. FAP was most significantly upregulated in kidney chromophobe (KICH) and cholangiocarcinoma (CHOL) tissues (**Figure 9c**). As shown in the forest plot in **Figure 9d**, the model genes had no obvious effect on the prognosis of most cancers. However, COL3A1 may have a negative impact on the prognosis of adrenocortical carcinoma (ACC), KICH, kidney renal clear cell carcinoma (KIRC), kidney renal papillary cell carcinoma (KIRP), brain lower grade glioma (LGG), etc. FBLN1 appeared to be a risk factor for thyroid carcinoma (THCA) and a protective factor for uveal melanoma (UVM). FAP showed a similar effect on the prognosis of THCA and UVM as FBLN1, and was also associated with favorable prognosis of pancreatic adenocarcinoma (PAAD). CYP7B1 was a negative prognostic factor for ACC, while GPC3 correlated with unfavorable prognosis of UVM and rectum adenocarcinoma (READ). ECM2 and AMPH were associated with the prognosis of KIRC, and AMPH was a protective factor in PAAD and sarcoma (SARC) (**Figure 9d**). We analyzed the expression levels of key model genes among the different immune subtypes, and detected similar trends. Specifically, the expression levels of COL3A1, CYP7B1, and GPC3 were generally low in the six immune subtypes (C1-C6), while the expression levels of ECM2, FAP, FBLN1, and AMPH were high (**Figure 9e**). The model genes exhibited strong positive correlations with both immune scores and stromal scores across the majority of cancers, and COL3A1 was significantly correlated with elevated immune scores and increased stromal scores in CHOL (**Figure 9f**). In contrast, the association between model genes and RNAss was predominantly negative, and that between model genes and DNAss was positive (**Figure 9g**).

### 3.8 GPC3 plays a tumorigenic role in osteosarcoma

**Figure 10:**
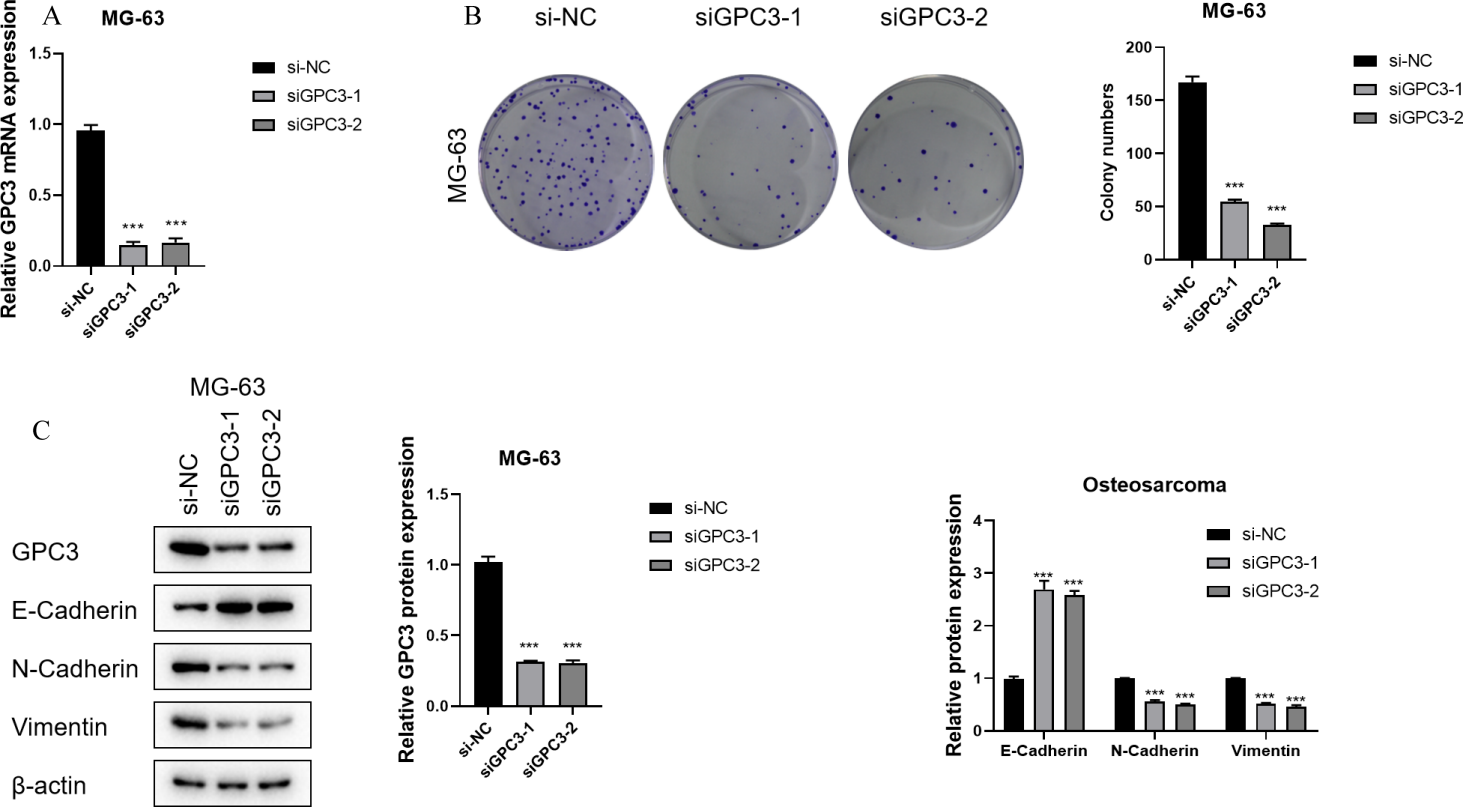
GPC3 functions as an oncogene in OS. **(A)** Bar graph showing GPC3 mRNA expression in different OS cell lines. **(B)** Bar graph showing GPC3 mRNA expression in OS cell lines after gene knockdown. **(C)** Line graph showing absorbance of U2OS cells in the CCK8 assay. *** typically indicates p < 0.001.

**Figure 11:**
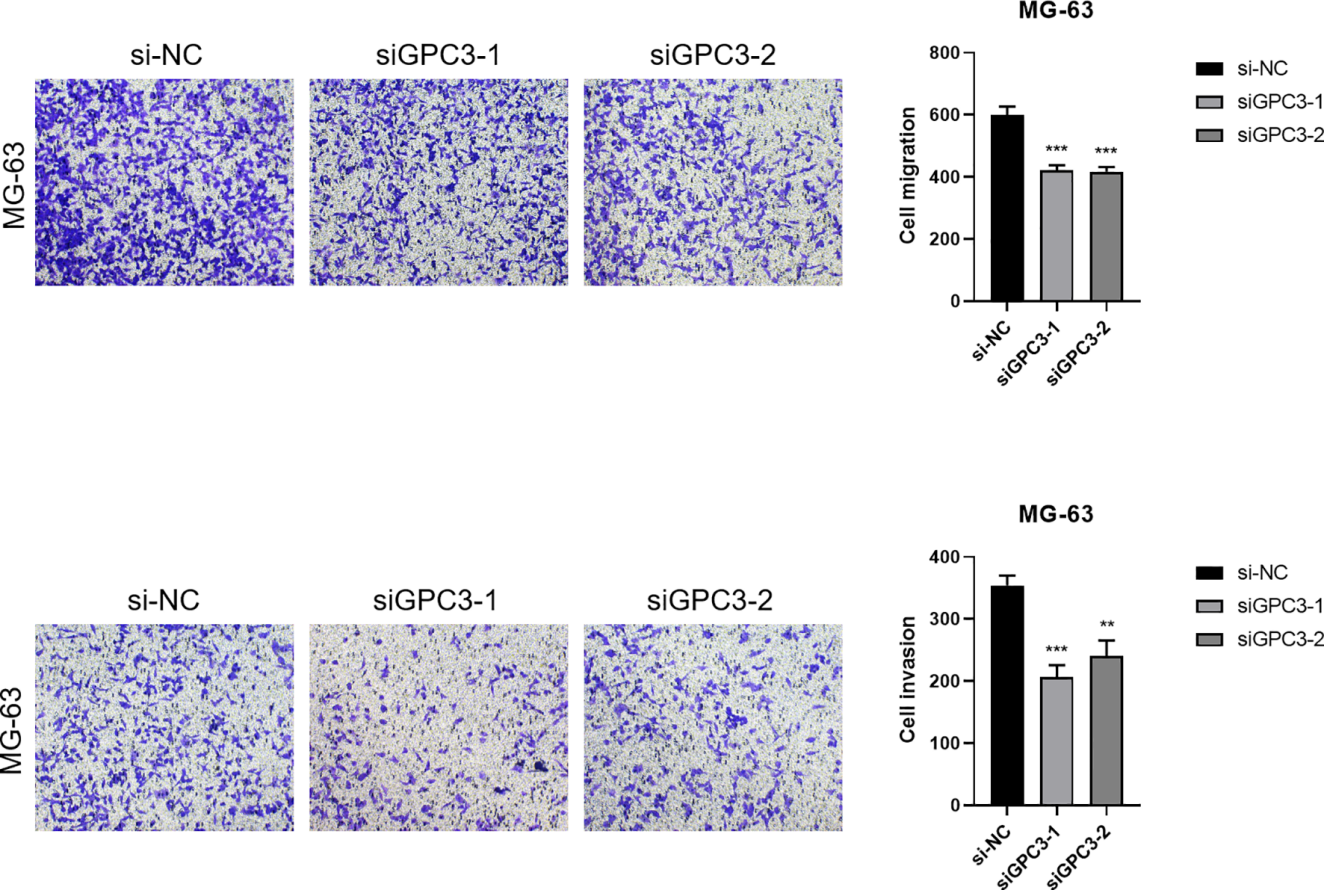
Transwell migration and invasion assays demonstrated that knockdown of GPC3 significantly inhibited the migration and invasive capacities of osteosarcoma cells compared to control groups. Quantification of migrated and invasive cells confirmed a substantial reduction following GPC3 silencing (*P* < 0.05). These findings suggest that GPC3 promotes both migration and invasion in osteosarcoma cells, highlighting its potential as a therapeutic target. *** indicates P < 0.001; ** indicates P < 0.01.

The construction of GPC3 si-RNA was performed and transfected into the MG-63 cell line to verify its knockdown efficiency. The results indicated that Si-GPC3–1 and Si-GPC3–1 exhibited good knockdown efficiency (p < 0.001, **Figure 10A**). The clone formation experiment verified the effect of GPC3 on the proliferation of the MG-63 cell line, showing that the knockdown of GPC3 significantly inhibited the proliferation of MG-63 (p < 0.001, **Figure 10B**). Western Blot experiments revealed significant changes in the expression of EMT-related pathway proteins following the knockdown of GPC3, with E-Cadherin protein levels significantly increased and N-Cadherin and Vimentin significantly decreased (p < 0.001, **Figure 10C**). Through the Transwell experiment, the proliferative ability of GPC3 on osteosarcoma cells was verified, revealing that after knocking down GPC3, the migration and invasion abilities of osteosarcoma cells significantly declined (**Figure 11**).

## 4 Discussion

OS is the most common primary malignant bone tumor in children and adolescents (2), and is routinely treated through surgery and chemotherapy. However, the 5-year survival rate is only about 60% (5, 6), and drops to 25% in patients with metastasis. The limited efficacy of current treatments warrants the development of novel therapeutic strategies to improve survival rates of OS patients (14). EMT is a process wherein cells lose their epithelial characteristics, and attain mesenchymal attributes like increased motility and invasiveness (15). It is involved in embryonic development, fibrosis, wound healing, inflammatory responses, and tumor metastasis (16), and may even contribute to MDR (17). The role of EMT is somewhat ambiguous in OS as it originates from mesenchymal cells. In fact, high expression of mesenchymal adhesion molecules can hinder the migration and spread of OS cells. Furthermore, BM-MSCs promote the migration and invasion of OS cells by upregulating cytokines such as IL-6 and IL-8 in the TME (21). Nevertheless, the mechanisms underlying EMT and MET in OS require additional investigation to address the heterogeneity among sarcoma subtypes, and discover new therapeutic strategies.

We identified two distinct clusters (C1 and C2) in the TARGET-OS cohort based on eleven EMT-related genes. C2 exhibited better prognosis compared to C1, while the latter had higher average age, and earlier onset of disease. The impact of age on the prognosis of OS remains uncertain, necessitating additional research. Interestingly, C1 showed greater infiltration of aDCs, B cells, CD8+ T cells, and T cells, which is often associated with a favorable prognosis, while C2 was associated with a more immunosuppressive landscape. The HALLMARK-related pathways were also downregulated in C2 compared to C1. Nevertheless, the prognostic impact of other clinical factors, such as the tumor type, stage, patients age, comorbidities, etc., cannot be excluded.

The EMT-related DEGs were divided into 10 gene modules through WCGNA. The brown module showed the strongest negative correlation with C2 and the strongest positive correlation with C1, indicating that the genes in this module may be potential prognostic biomarkers and therapeutic targets for OS. The hub genes of the brown module were mainly associated with the ECM, which not only provides structural support to cells, but also regulates cellular communication, migration, adhesion, proliferation and differentiation. Furthermore, ECM proteins such as collagen, fibronectin, laminin, and proteoglycans have been linked to the metastasis of OS cells (22). We further extracted seven candidate genes from the intersection of hub genes and DEGs to establish a prognostic model for OS. The model was applied to the TARGET-OS, GSE21257 and GSE16091 datasets, and each cohort was classified into high-risk and low-risk categories. The high-risk group had worse prognosis compared to the low-risk group across all cohorts, which was indicative of the predictive ability of the model. Furthermore, four common immune regulatory factors and effector genes linked to tumor-related immune cells were observed to be expressed at heightened levels in the low-risk group in the TARGET-OS cohort, which suggests a relationship between the overall survival risk score and the immune profile. It remains to be ascertained whether these immune-related genes can predict patients prognosis and response to immunotherapy. Furthermore, the anti-cancer immune cycle scores in the low-risk group exceeded that in the high-risk group, which is significant for cancer diagnosis, treatment, and prognosis. Consistent with this, the low-risk group had a greater abundance of anti-tumor immune cells, whereas the high-risk group had a more immunosuppressive profile. The greater understanding of the immune characteristics of different risk groups can aid in the development of personalized treatment methods.

According to the TIDE scores, the high-risk group showed a greater probability of immune escape compared to the low-risk group. Thus, patients in the high-risk group would likely respond poorly to immune checkpoint inhibitors and other immunotherapies. Furthermore, the CAF score, exclusion score, dysfunction score, and TAM-M2 score were all elevated in the high-risk group compared to that in the low-risk group. This is indicative of lower infiltration of fibroblasts and T cells, and a higher abundance of M2 macrophages in the high-risk group, which correspond to an immunosuppressive TME that is less responsive to immunotherapy. In contrast, the low-risk group had elevated scores for CD8 T cells, GEP, TLS, and Merck18. The increased infiltration of CD8+ T cells and other lymphocytes in the low-risk cohort suggested enhanced immune activity that may results in favorable outcomes. The immune characteristics associated with the two risk categories can serve as valuable indicators for evaluating patient prognosis and response to immunotherapy, and offer insights into the immune escape mechanisms employed by OS cells. We applied the EMT model to six independent immunotherapy cohorts, and found that the high-risk group had worse prognosis compared to the low-risk group in every dataset. In addition, the risk scores of patients that responded poorly to immune checkpoint inhibitors were also significantly higher than that of responders, thus confirming that EMT may be associated with poor prognosis in OS. Furthermore, the EMT signature can be used to select patients that are more likely to respond to immunotherapy, potentially resulting in improved treatment outcomes and survival rates.

We identified 7 major cell categories and 19 cell groups from the scRNA-Seq data of six OS samples. The osteoblasts and MSCs shared markers including COL1A1, CPE, and COLA1A2, which offers insights into the differentiation of osteoblasts and the regulatory mechanisms. Furthermore, monocytes and osteoblasts were the predominant populations, and may play an important role in the pathogenesis of OS. The osteoblasts were divided into the high-score and low-score subgroups according to the median EMT score. The low-score cells were enriched in pathways related to adhesion, cytoskeleton, and Pl3K-Akt signaling, while pathways related to metabolism and tumor development were upregulated in the high-score cells. The potential of these pathways as therapeutic targets for OS treatment need to be explored further. Dimensionality reduction clustering of the osteoblast population revealed seven subpopulations, of which OS_C3 was identified to be in the terminal stage of development due to its lowest differentiation potential, and the OS_C5 was identified as a stem cell population due to its highest differentiation potential. The C3 subpopulation was most abundant, indicating that it may be involved in the development of OS, and even have diagnostic or therapeutic value. Through hdWGCNA, we obtained nine modules from the C3 subpopulation, and clustered them to determine the distribution and mutual relationships of the cells. OS_C3-M1 and OS_C3-M2 showed high similarity, as did OS_C3- M4 and OS_C3-M5. In addition, OS_C3-M3 has higher similarity with OS_C3-M8 and OS_C3-M9. The hub genes of these modules showed strong interactions, and were mainly associated with metabolism, structural integrity, proliferation, differentiation, adhesion, migration, immune responses, inflammatory reactions, and signal transduction. The varying expression levels of these genes may influence the progression of OS, indicating their potential as therapeutic targets.

Among the seven model genes, FBLN1, FAP, and ECM2 exhibited the highest expression levels in the OS samples. FBLN1 is an extracellular glycoprotein involved in the regulation of cell morphology, growth, adhesion, and movement. It functions as a tumor suppressor in prostate cancer and breast cancer, and the inactivation of FBLN1 has been linked to the progression of gastric cancer (23). FAP is a surface serine protease that is upregulated in reactive stromal fibroblasts, and has been detected in 90% of cancers (24). EMC2, a member of the endoplasmic reticulum membrane protein complex (EMC) family, is associated with ferroptosis and highly expressed in tumors like COAD, LUSC, and BRCA (25). FAP and EMC2 showed the strongest correlation, suggesting similar functions in the formation, remodeling, and degradation of the ECM. Pan-cancer analysis of these genes showed high expression of all seven genes in CHOL, and upregulation of EMC2 in most tumors, including OS. The remodeling and breakdown of ECM are conducive to the invasion and metastasis of cancer cells. Furthermore, FBLN1, FAP, ECM2, and AMPH were upregulated in the immune subtypes, which is indicative of the complex interactions among the immune cells and other components in the TME. The above genes may be considered as potential therapeutic targets and prognostic markers for OS< and warrant further investigation. COL3A1 was strongly correlated with the infiltration of immune cells and matrix cells in CHOL, while GPC3 and AMPH were strongly correlated with the immune infiltration in UCS. Furthermore, FAP and AMPH were related to stromal infiltration and stemness in most tumors, indicating a key role in tumor occurrence and development.

COL3A1, also known as type III collagen, is abundant in blood vessels (26) and plays a key role in breast cancer metastasis. Fibroblasts expressing high levels of EMP1 and COL3A may be involved in the metastatic processes of breast cancer, kidney cancer, and prostate cancer (27). GPC3 is a cell surface oncofetal protein that is overexpressed in 70% of hepatocellular carcinoma (HCC) cases, and promotes tumor growth by regulating the Wnt/Frizzled signaling complex (28). AMPH is predominantly expressed in neuronal synapses, and may play a role in exocytosis and in the dynamic organization of membrane-associated cytoskeleton. Aberrant expression of AMPH has been linked to dysregulated actin distribution, which affects entry into quiescence (29). CYP7B1, also known as cytochrome P450, acts on hydroxylated steroids such as dehydroepiandrosterone, 25-hydroxycholesterol, and 27-hydroxycholesterol. Abnormal expression of CYP7B1 may lead to neonatal liver failure and progressive neurodegeneration in adults (30). The function of GPC3 in OS is ambiguous at present. Knocking down GPC3 in OS cell lines inhibited their proliferation, indicating that GPC3 has an oncogenic function in OS.

## 5 Conclusion

We established a prognostic model for OS based on the genes related to EMT. The EMT-based model predicted the prognosis, immune landscape, and immunotherapy responses on OS patients, and may prove to be valuable for the development of personalized treatment strategies.

## Data Availability

The raw data supporting the conclusions of this article will be made available by the authors, without undue reservation.
